# Heparanase as an Additional Tool for Detecting Structural Peculiarities of Heparin Oligosaccharides

**DOI:** 10.3390/molecules24234403

**Published:** 2019-12-02

**Authors:** Anna Alekseeva, Elena Urso, Giulia Mazzini, Annamaria Naggi

**Affiliations:** 1 Centro Alta Tecnologia Istituto di Ricerche Chimiche e Biochimiche G. Ronzoni Srl, via G. Colombo 81, 20133 Milan, Italy; g.mazzini@smatteo.pv.it (G.M.); naggi@ronzoni.it (A.N.); 2 Istituto di Ricerche Chimiche e Biochimiche G. Ronzoni, via G. Colombo 81, 20133 Milan, Italy

**Keywords:** heparanase, heparin lyases, low molecular weight heparins, bovine mucosal heparin, antithrombin binding site, mass spectrometry

## Abstract

Due to the biological properties of heparin and low-molecular-weight heparin (LMWH), continuous advances in elucidation of their microheterogeneous structure and discovery of novel structural peculiarities are crucial. Effective strategies for monitoring manufacturing processes and assessment of more restrictive specifications, as imposed by the current regulatory agencies, need to be developed. Hereby, we apply an efficient heparanase-based strategy to assert the structure of two major isomeric octasaccharides of dalteparin and investigate the tetrasaccharides arising from antithrombin binding region (ATBR) of bovine mucosal heparin. Heparanase, especially when combined with other sample preparation methods (e.g., size exclusion, affinity chromatography, heparinase depolymerization), was shown to be a powerful tool providing relevant information about heparin structural peculiarities. The applied approach provided direct evidence that oligomers bearing glucuronic acid–glucosamine-3-*O*-sulfate at their nonreducing end represent an important structural signature of dalteparin. When extended to ATBR-related tetramers of bovine heparin, the heparanase-based approach allowed for elucidation of the structure of minor sequences that have not been reported yet. The obtained results are of high importance in the view of the growing interest of regulatory agencies and manufacturers in the development of low-molecular-weight heparin generics as well as bovine heparin as alternative source.

## 1. Introduction

Heparin is a widely used anticoagulant and antithrombotic drug which structurally represents a highly sulfated linear polydisperse glucosaminoglycan (GAG) that primarily consists of a repeating 2-*O*-sulfated iduronic acid (I_2S_) and glucosamine-N,6-*O*-disulfate (A_NS6S_). Heparin structural complexity is further increased by the presence of glucuronic acid (G) and *N*-acetyl-glucosamine (A_NAc_) as well as other minor structures, such as 2-*O*-sulfated glucuronic acid (G_2S_), linkage region (LR), 3-*O*-sulfated glucosamine-*N*-sulfate (A_NS3S(6S)_) [1,2,3]. The latter, when preceded by G, is a marker of antithrombin binding region (ATBR) (A_NX6S_–G–A_NS3S6S_–I_2S_–A_NS6S_; X = Ac or SO_3_) essential for high anticoagulant and antithrombotic activity of heparin [4,5,6]. While the presence of the –G–A_NS3S6S_– fragment is mandatory for the binding, the sulfation/acetylation pattern of the other residues can vary [6,7]. In the case of enzymatically or chemically generated low-molecular-weight heparins (LMWHs), unnatural residues at reducing (RE) and nonreducing end (NRE) introduce additional features in its complex structure [2,8,9].

The variety of biological properties of heparin/LMWH is strongly related to its high sulfation degree and structural microheterogeneity, determining binding with numerous proteins and other biomolecules. From the other side, their structural complexity together with high molecular weight significantly complicate their analysis. In fact, heparin/LMWH structural elucidation cannot be performed using a single method but requires the combination of different orthogonal methods, in particularly high-resolution NMR and mass spectrometry (MS) [7,10,11,12]. Particularly, high-resolution MS allowed the achievement of previously unthinkable sensitivity and specificity in identification of heparin-related oligosaccharides and their building blocks [11,12,13,14,15,16]. To overcome the problem of high molecular weight and microheterogeneity, enzymatic degradation with heparinases followed by a refined LC-MS analysis is often involved in the structural characterization scheme [10,17,18,19,20]. It has been extensively reported that heparinases I and III show high specificity by cleavage of highly sulfated and undersulfated regions, respectively, while heparinase II displays a broad range of substrate specificity [17,18,19]. Particularly, heparinase I cleaves the linkage A_NS3X6X_–I_2S_ (X = H or SO_3_), heparinase III cleaves A_NAc6X_–I and A_NS6X_–G (X = H or SO_3_), while heparinase II essentially cleaves glycosidic linkages containing both 2-*O*-sulfated and nonsulfated uronic acids. Nowadays, the use of heparinases is recognized to be a valuable method to obtain patterns characteristic for different animal sources, to determine minor unusual sequences or contaminating species, and to compare different heparin/LMWH batches for sameness studies [21]. However, despite relevant structural information it can provide about heparin composition, heparinase depolymerization is often not sufficient to elucidate specific sequences that are resistant to these enzymes. Particularly, none of them can cleave the A–G linkage when followed by A_NS3S(6S)_ within ATBR [7,10]. The resistance of tetrasaccharides arising from the ATBR to heparinases, together with the absence of their standards, does not allow determination of their sequences and consequent description of the composition of ATBR variants within heparin samples without time-consuming isolation steps.

In this work we propose a combined approach that implicates the use of heparanase as heparin degrading enzyme prior to LC-MS analysis, when heparinase digestion did not provide sufficient structural information about heparin sequences. Heparanase is a β-d-endoglucuronidase [22,23] confined inside specific cells under the normal conditions but found in the circulation of patients with most oncological or inflammation diseases [24,25]. Extracellular heparanase is thought to be a critical player in tumor growth and progression, since it may alter the integrity of extracellular matrix through the release of growth factors from heparan sulfate (HS) storage sites. It acts through hydrolytic action of the linkage between the anomeric position of G and the following residue, while its specificity is dependent on the substrate size and sulfation pattern with the preference for the longer sulfated chains [26,27,28]. Interestingly, the presence of an A_NS3S(6S)_ at the RE of the site of substrate cleavage exhibits a promoting effect in relatively lowly sulfated sequences but inhibits the cleavage of highly sulfated structures [26]. On the other hand, ATBR-related sequence of synthetic pentasaccharide A_NS6S_–G–A_NS3S6S_–I_2S_–A_NS6S_^OMe^ (Fondaparinux^®^) was shown to be susceptible to heparanase by Bisio et al. [29]. This susceptibility was used for the development of a sensitive LC-MS approach for measuring heparanase activity by using the synthetic pentasaccharide as a substrate. Mao et al. [28] used heparanase depolymerization prior to a limited digestion by heparinase III to generate odd-numbered HS oligosaccharides to further explore the corresponding heparanase cleavage sites and enzyme substrate specificity. In spite of the mentioned studies, heparanase enzymatic properties have not been exploited yet for the in-depth structural characterization of heparin oligosaccharides.

In the present study we exploited heparanase specificity towards G–A_NS3S(6S)_-containing sequences in order to show its potential application for structural elucidation of particular heparin-related oligosaccharide sequences. Here, we report two particular examples of its implication for heparin structural analysis. Firstly, heparanase was used to assert the structure of two major components of a dalteparin octasaccharide fraction, previously only tentatively assigned to I_2S_–A_NS6S_–I_2S_–A_NS6S_–I_2S_–A_NS6S_–I_2S_–aM.ol_6S_ and G–A_NS3S6S_–I_2S_–A_NS6S_–I_2S_–A_NS6S_–I_2S_–aM.ol_6S_ (where aM.ol is a 2,5-anhydromannitol) [12]. The variety of terminal residues of LMWHs represents one of the important structural signatures of these drugs. The presence of G–A_NS3S6S_– moieties at the NRE of dalteparin oligomers would provide additional information on the depolymerization process.

Secondly, the heparanase-based strategy was extended to the structural elucidation of tetrasaccharides of bovine mucosal heparin (BMH) resistant to heparinases. Structural analysis of bovine-derived heparin is of particular interest because of the attempt of its reintroduction to the US market in order to diversify heparin sources and guarantee its supply [30].

Several strategies using heparinases are currently employed to characterize disaccharide building blocks of heparins. In the present study we show that the use of heparanase can add value to the traditional heparinase-based strategy. When applied to a BMH fraction with high affinity towards antithrombin (HA fraction), it allowed the identification of some new ATBR-related tetrasaccharides in addition to the already reported structures [7,10,16,31,32,33]. Their structure and distribution reflect the structural heterogeneity of ATBR sequences strongly associated with heparin biological activity.

Notably, the results reported hereafter provide some new insights about heparanase substrate specificity.

## 2. Results and Discussion

### 2.1. Identification of G–A_NS3S6S_ Sequence at the NRE of Dalteparin Octasaccharides

As mentioned above, our previous studies [12] suggest that some major components of dalteparin bear a G–A_NS3S6S_ unit at their NRE. This was also consistent with the recently published study showing the presence of at least two U8,11,0–aM.ol isomers [34] as well as our previously work on glycol-split LMWHs, where the presence of nonsulfated uronic acids at the NRE of fully sulfated oligomers was hypothesized [35]. Notably, this phenomenon was observed for each size fraction, making these isomers characteristic structural features of dalteparin and sensitive markers of depolymerization process conditions.

We initially attempted to isolate U8,11,0–aM.ol isomers by ion pair reversed-phase (IPRP) chromatography for their detailed NMR study. Despite IPRP UHPLC separation mode having been previously shown to provide a good resolution between these isomers [12], the method transfer to a preparative HPLC scale caused a dramatic decrease in their separation. Moreover, their isolation was complicated by difficulties associated with signal monitoring due to low UV absorbance of saturated oligosaccharides. Alternatively, the combination of enzymatic methods, the UHPLC separation mode and the introduction of the on-line collision ion dissociation (CID) MS/MS analysis did provide some relevant data for the elucidation of the isomeric structures.

**Figure 1:**
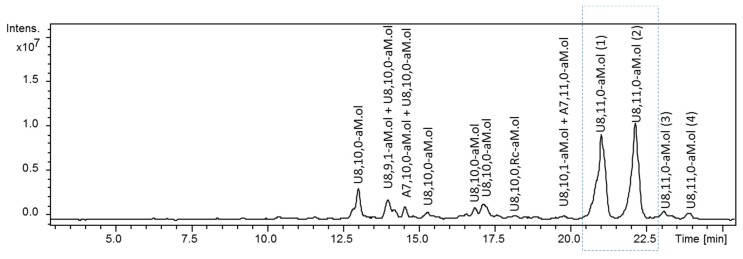
UHPLC/ESI-MS chromatograms of octasaccharide dalteparin fraction isolated by SEC. Two major U8,11,0-aM.ol isomers of interest are shown in the rectangular frame. Monoisotopic *m/z* values for prevalent ion forms: U8,10,0–aM.ol, *m/z* 845.135 ([M − 3H + 3DBA]^3−^); U8,9,1–aM.ol, *m/z* 875.537 ([M − 3H + 4DBA]^3−^); A7,10,0–aM.ol, *m/z* 786.458 ([M − 3H + 3DBA]^3−^); U8,10,0,Rc–aM.ol, *m/z* 883.182 ([M − 3H + 4DBA]^3−^); A7,11,0–aM.ol, *m/z* 856.160 ([M − 3H + 4DBA]^3−^); U8,10,1–aM.ol, *m/z* 902.189 ([M − 3H + 4DBA]^3−^); U8,11,0–aM.ol, *m/z* 914.838 ([M − 3H + 4DBA]^3−^).The abbreviation system includes, in order, the number of monosaccharide residues, sulfate groups, and *N*-acetyl groups. Symbols U and A were added to indicate a saturated uronic acid and a glucosamine unit, respectively, at the NRE, while aM.ol and Rc indicate 2,5-anhydro-mannitol at the RE and ring-contracted residues [12,35], respectively.

**Figure 2:**
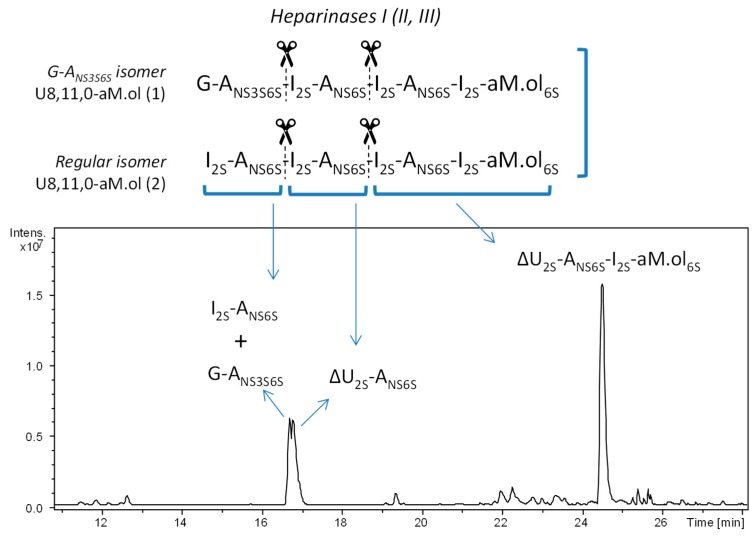
UHPLC/ESI-MS chromatogram of octasaccharide dalteparin fraction exhaustively digested with heparinases I, II and III prior to the analysis. Monoisotopic *m/z* values for major digestion products: U2,3,0, *m/z* 593.975 ([M – H]^–^) assigned to I_2S_–A_NS6S_ and G–A_NS3S6S_; ΔU2,3,0, *m/z* 575.964 ([M − H]^−^) assigned to ΔU_2S_–A_NS6S_; ΔU4,5,0–aM.ol, *m/z* 528.480 ([M − 2H]^2−^) assigned to ΔU_2S_–A_NS6S_–I_2S_–aM.ol_6S._ The abbreviation system includes, in order, the number of monosaccharide residues, sulfate groups, and *N*-acetyl groups. Symbols ΔU and U were added to indicate a 4,5-unsaturated uronic acid, saturated uronic acid and a glucosamine unit, respectively, at the nonreducing end (NRE), while aM.ol indicates 2,5-anhydro-mannitol residue at the reducing end (RE).

The LC-MS analysis did show that the two main components of octasaccharide fraction are the isomeric undecasulfated octasaccharides U8,11,0–aM.ol bearing aM.ol at the RE (Figure 1). The fragment distribution arising from the action of heparinases I, II and III on dalteparin octasaccharide fraction (Figure 2) was consistent with the hypothesis of the structure of two isomeric octasaccharides I_2S_–A_NS6S_–I_2S_–A_NS6S_–I_2S_–A_NS6S_–I_2S_–aM.ol_6S_ and G–A_NS3S6S_–I_2S_–A_NS6S_–I_2S_–A_NS6S_–I_2S_–aM.ol_6S_. Among the major components, the exhaustive heparinase digestion generated an unsaturated tetrasaccharide ΔU4,5,0–aM.ol (–I_2S_–A_NS6S_–I_2S_–aM.ol_6S_) resistant to heparinase due to the presence of unnatural RE [36] as well as unsaturated disaccharide ΔU2,3,0, arising from the internal –I_2S_–A_NS6S_– sequences accompanied by two saturated disaccharides U2,3,0 with three sulfate groups, whose formation was consistent with the presence of I_2S_–A_NS6S_– and G–A_NS3S6S_– at the NRE of the two octamers. Notably, the absence of tetrasulfated disaccharides, with neither saturated U2,4,0 nor unsaturated ΔU2,4,0 [7,10] found in heparinase digest (Figure 2), suggests that trisulfated glucosamine is preceded by a nonsulfated uronic acid, but not an I_2S_ residue.

Since an efficient sugar ring fragmentation cannot retain the labile sulfate groups, the MS/MS fragmentation in CID mode precludes the exact structure identification. At any rate, the MS/MS experiments of these disaccharides produced some useful fragments arising from intra-ring cleavages A, B, X, Y and Z [37,38]. The fragment ^0,2^X_2_ appears more intense in the first isomer than the second one, while the ion ^0,2^A_2_ at *m/z* 198.99 was detected only in the MS/MS spectrum of the first isomer. This observation, in agreement with the known behavior that the ^0,2^A ion fragments are favored by a more sulfated glucosamine [39], suggests that the first isomer U2,3,0 corresponds to a nonsulfated uronic acid U followed by A_NS3S6S_ unit. In addition, the ion B_1_ – CO_2_ (+1 SO_3_) at *m/z* 210.99 arising from the 2-*O*-sulfated uronic acid and detected only in the fragmentation spectrum of the second isomer, confirmed the presence of the I_2S_–A_NS6S_– fragment at the NRE disaccharide within the corresponding octamer (Table 1).

**Figure 3:**
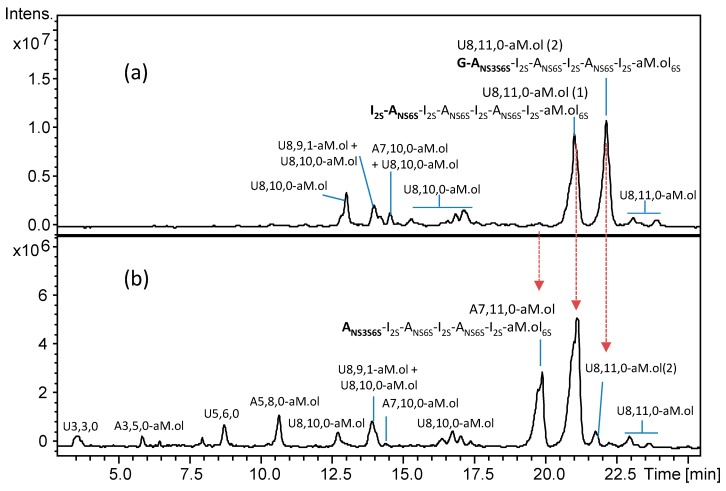
UHPLC/ESI-MS chromatograms of octasaccharide dalteparin fraction isolated by SEC (**a**) before and (**b**) after heparanase digestion. Monoisotopic *m/z* values for prevalent ion forms: U3,3,0, *m/z* 384.500 ([M − 2H]^2−^); A3,5,0–aM.ol, *m/z* 449.469 ([M − 2H + DBA]^2−^); U5,6,0, *m/z* 737.561 ([M − 2H + DBA]^2−^); A5,8,0–aM.ol, *m/z* 931.683 ([M − 2H + 3DBA]^2−^); U8,10,0–aM.ol, *m/z* 845.135 ([M − 3H + 3DBA]^3−^); U8,9,1–aM.ol, *m/z* 875.537 ([M − 3H + 4DBA]^3−^); A7,10,0–aM.ol, *m/z* 786.458 ([M − 3H + 3DBA]^3−^); U8,10,0,Rc–aM.ol, *m/z* 883.182 ([M − 3H + 4DBA]^3−^); A7,11,0–aM.ol, *m/z* 856.160 ([M − 3H + 4DBA]^3−^); U8,10,1–aM.ol, *m/z* 902.189 ([M − 3H + 4DBA]^3−^); U8,11,0–aM.ol, *m/z* 914.838 ([M − 3H + 4DBA]^3−^). The abbreviation system includes, in order, the number of monosaccharide residues, sulfate groups, and *N*-acetyl groups. Symbols U and A were added to indicate a saturated uronic acid and a glucosamine unit, respectively, at the NRE.

The structural detail that allowed to complete the identification of the structure G–A_NS3S6S_–I_2S_–A_NS6S_–I_2S_–A_NS6S_–I_2S_–aM.ol_6S_ emerged after the enzymatic cleavage of dalteparin octasaccharide fraction with heparanase. The LC-MS analysis of the octasaccharide fraction before and after heparanase treatment showed the simultaneous resistance of the isomer U8,11,0–aM.ol eluted at RT 21.1 min (*m/z* 914.838 of [M – 3H + 4DBA]^3–^ ion form) and the disappearance of the isomer eluted at RT 22.2 min (*m/z* 914.838 of [M – 3H + 4DBA]^3–^ ion form) and, importantly, the appearance of a new peak at RT 19.9 min with the *m/z* 856.160 attributed to a [M – 3H + 4DBA]^3–^ ion of heptasaccharide A7,11,0–aM.ol (Figure 3). Relying on the known specificity of heparanase towards G–A_NS_ linkages [27], the formation of a heptasaccharide with a glucosamine at the NRE and the same sulfation degree as the U8,11,0–aM.ol directly indicates that this heptamer is a heparanase-generated depolymerization product and the isomer, susceptible to heparanase, contained a nonsulfated G at its NRE. An increase of monosaccharide A_NS3S6S_ peak, observed after further digestion with heparinases (Figure S1), proved that this glucosamine is at the NRE end of the heparanase-generated heptamer.

The obtained data provide the evidence that heparanase can cleave even a single monosaccharide from the NRE, opening new questions about the heparanase mechanism of action. Despite being in accord with its mechanism of action consisting in the hydrolytic cleavage from the NRE [27,40], monosaccharide release by an endo-enzyme was quite unexpected. To our knowledge, monosaccharide digestion product has not been reported yet.

Together with the prevalent U8,11,0–aM.ol isomers, their minor isomers eluted between 22.5 and 24 min are believed to bear G–A_NS3S6S_ moiety within their chains accordingly to Bisio et al.: I_2S_–A_NS6S_–G–A_NS3S6S_–I_2S_–A_NS6S_–I_2S_–aM.ol_6S_ and I_2S_–A_NS6S_–I_2S_–A_NS6S_–G–A_NS3S6S_–I_2S_–aM.ol_6S_ [12]. Heparanase only partially hydrolyzed these isomers, however, the appearance of tri- and pentasaccharide pairs (U3,3,0/A5,8,0–aM.ol and U5,6,0/A3,5,0–aM.ol, respectively, Figure 3) is in accordance with the proposed structures (Figure 3).

It is worth also noting that the use of heparanase generated additional information about heterogeneity level of heparins. Even if it may be complicated to unambiguously determine the digestion products of each oligomer when it comes to minor components, the heparanase-generated LC-MS profiles may represent characteristic fingerprints for these complex drugs.

Even more interestingly, it is worth discussing the possible formation of oligomers bearing G–A_NS3S6S_ at their NRE. Their relatively high abundance and above-mentioned presence in all the size fractions of commercial dalteparin samples analyzed in our laboratory make them an important structural signature of this LMWH. It is well established that the nitrous acid depolymerization used in dalteparin production occurs at the level of N-sulfated glucosamine and an uronic acid, where N-sulfation represents mandatory structural factor [41]. Thus, the isomers containing G–A_NS3S6S_ at the NRE detected in dalteparin arise from the depolymerization of the N-sulfated variant of ATBR (–A_NS6S_–G–A_NS3S6S_–I_2S_–A_NS6S_–), but not its N-acetylated variant (–A_NAc6S_–G–A_NS3S6S_–I_2S_–A_NS6S_–), predominant in porcine mucosa heparin.

### 2.2. Structural Elucidation of Heparin G–A_NS3S6S_ Containing Oligomers Resistant to Heparinase Action

Furthermore, the effectiveness of the heparanase-based strategy was extended to the structural elucidation of tetrasaccharides of bovine heparin, resistant to exhaustive digestion by heparinases and, consequently, associated with the presence of G–A_NS3S(6S)_ fragment at the RE [7,10,31,32,33]. To specifically explore the species containing the ATBR, the HA fraction of a BMH, enriched in these sequences by affinity chromatography fractionation, was investigated. A multistep salt gradient was applied as described in Section 3 and a fraction, eluted with 3M sodium chloride and expected to possess the highest affinity to AT, was further studied.

LC-MS analysis of these oligomers, subjected to exhaustive heparinases digestion (Figure S2a) showed, as expected, that the main digestion product is represented by the trisulfated disaccharide ΔU_2S_–A_NS6S_ and the less intense disulfated ones (ΔU–A_NS6S_ and ΔU_2S_–A_NS_) reflecting the regular I_2S_–A_NS6S,_ I/G–A_NS6S_ and I_2S_–A_NS_ sequences, respectively. Monosulfated and monoacetylated disaccharides were detected as minor components in addition to a few linkage region fragments: the intact linkage region (LR: ΔU–Gal–Gal–Xyl–Ser), its acetylated form (LR-Ac: ΔU–Gal–Gal–Xyl–Ser–COCH_3_) and the oxidated form (LR-ox: ΔU–Gal–Gal–Xyl–CH_2_COOH). It can be noted that, even if the serine acetylation has already been observed by Chen et al. in a porcine sample [16], we have found this fragment only in the bovine ones (unpublished data).

More interestingly, the oligosaccharides region showed several species that are resistant to exhaustive heparinase cleavage (Figure S3a). Among those, the major tetrasaccharides generated from the AT-binding site (ΔU4,3,1 (ΔU–A_NAc6S_–G–A_NS3S_); ΔU4,4,1 (ΔU–A_NAc6S_–G–A_NS3S6S_); ΔU4,5,0 (1) (ΔU–A_NS6S_–G–A_NS3S6S_); ΔU4,5,0 (2) (ΔU_2S_–A_NS_–G–A_NS3S6S_) and ΔU4,6,0 (ΔU_2S_–A_NS6S_–G–A_NS3S6S_)) have been previously reported [7]. The third detected isomer, ΔU4,5,0 (3), was also observed in bovine heparin in the previously published work, however, its structure has not been assigned.

Notably, we found other minor tetrasaccharides attributed to ΔU4,4,0 and an additional isomer of ΔU4,4,1 (eluted between 33.5 and 35 min) as well as ΔU4,5,1 (eluted at about 37.5 min), whose generation and structure have not been reported previously. More unexpectedly, very lowly abundant hexasaccharides with *m/z* 913.6 (z = −2) and *m/z* 999.2 (z = −2), attributed to ΔU6,7,0 (unsaturated hexasaccharide with seven sulfate groups) and ΔU6,7,1 (unsaturated hexasaccharide with seven sulfate groups and one acetyl group), respectively, were detected (Figure 5a and Figure S3). The presence of three positional isomers of ΔU6,7,0 was exclusively observed in HA bovine heparin, while only a heparinase-resistant ΔU6,6,1 hexamer has been previously found in HA porcine heparin fraction [7]. These findings are both in agreement with higher abundance of N-acetylated ATBR variant in porcine heparin.

**Figure 4:**
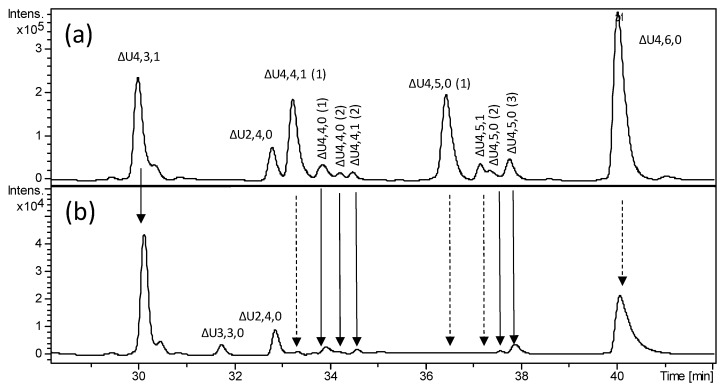
LC-MS profile of bovine heparin fraction with high affinity towards antithrombin and mass signal assignment of the main components. Expanded chromatogram focused on oligosaccharides region affected by the heparanase action: (**a**) heparinase digest of BMH; (**b**) heparanase digestion of heparinase digest. Dashed arrow—susceptibility toward heparanase action. Solid arrow—some resistance toward heparanase action. The abbreviation system includes, in order, the number of monosaccharide residues, sulfate groups, and *N*-acetyl groups. Symbol ΔU was added to indicate a 4,5-unsaturated uronic acid at the NRE.

To record new structural information about the heparinase-resistant tetra- and hexasaccharides, an attempt was performed by using the new strategy consisting of the heparanase addition to previous heparinase digest. Addition of heparanase did cause a significant change in LC-MS profile, especially, in its oligosaccharide region (Figure 4a,b). Interestingly, some of the heparinase-resistant oligomers appeared resistant to heparanase, while some of them totally disappeared from chromatogram, suggesting the presence of structural differences around the G–A_NS3S6S_ linkage that were associated with different sulfation patterns.

Examining the major tetrasaccharides, ΔU4,3,1 (ΔU–A_NAc6S_–G–A_NS3S_) resulted as the most resistant to heparanase action, while ΔU4,4,1 (ΔU–A_NAc6S_–G–A_NS3S6S_) and the first isomer of ΔU4,5,0 (ΔU–A_NS6S_–G–A_NS3S6S_) seem to totally disappear. These tetrasaccharides share a very similar sequence around the glucuronic acid, therefore the unexpected resistance of one of them is supposed to be related to small structural differences among their structures. The tetrasaccharide ΔU4,6,0 (ΔU_2S_–A_NS6S_–G–A_NS3S6S_) showed a significant susceptibility toward the heparanase although it has not completely digested, in agreement with some heparanase resistance (as mentioned above) displayed by the –G–A_NS3S6S_– intrachain sequence of minor U8,11,0–aM.ol isomers detected in dalteparin. The partial resistance of this tetrasaccharide can be explained by the inhibitory effect in a highly sulfated sequence, as mentioned in the introduction [26], while the others are believed to be resistant because of low sulfation around the cleavage site.

It can be noticed that the resistant ΔU4,3,1 (ΔU–A_NAc6S_–G–A_NS3S_) shows the presence of A_NS3S_ in place of A_NS3S6S_ located at the RE of ΔU4,4,1 (ΔU–A_NAc6S_–G–A_NS3S6S_) which, in contrast, resulted as extensively digested.

In view of this observation, the revealed resistance of ΔU4,3,1 can be attributed to the lack of 6-*O*-sulfation at the RE, in accordance with previously published studies [26,27], and allows to explain the presence of the unknown ΔU4,4,1 (2) and ΔU4,5,0 (3) isomers (eluting at ~34.5 and ~38 min, respectively; Figure 4b) whose heparanase resistance is believed to be associated with the lack of 6-*O*-sulfation at the RE within the following structures ΔU_2S_–A_NAc6S_–G–A_NS3S_ and ΔU_2S_–A_NS6S_–G–A_NS3S_. Based on the same considerations, both the two unknown isomers of ΔU4,4,0 shown to be little susceptible to heparanase, were assigned to ΔU_(2X)_–A_NS(6X)_–G–A_NS3S_ (X = OH or SO_3_).

Furthermore, the resistance toward heparanase exhibited by the tetrasaccharide ΔU4,5,0 (2) (ΔU_2S_–A_NS_–G–A_NS3S6S_), whose structure was previously reported [7], suggests that the lack of 6-*O*-sulfation on the glucosamine preceding the G–A_NS3S6S_ linkage can also affect the active site recognition.

Among the new fragments arising from the hydrolytic cleavage of the G–A bond (Figure 4b and Figure S3), odd and even species were detected (Figure S3): unsaturated odd species in whose structures the reducing end G arises from the NRE of the G–A linkage (e.g., the unsaturated trisaccharides ΔU3,1,1; ΔU3,2,0; ΔU3,3,0) and saturated hexosamine residues (e.g., A1,3,0) from the RE of the G–A linkage.

Based on the peculiar heparanase specificity [26] and taking into account both the resistance of a few tetrasaccharides and the generation of specific enzymatic fragments, further interesting insights have emerged, as reported in Table 2. Particularly, apart from the previously identified tetrasaccharides, a structure was proposed for each of the species so far not identified. Specifically, the structures of tetrasaccharides showing some resistance to heparanase due to the lack of 6-*O*-sulfation at their RE (ΔU4,3,1, ΔU4,4,0 (1) and (2), ΔU4,4,1 (2) and ΔU4,5,0 (3), (Table 2)) appear to be supported by an extremely small amount of an A_NS3S_ monomer.

The proposed elution order of the tetrasaccharides ΔU4,4,0 (1) and (2) (Table 2) is supported by previous evidence that the chromatographic separation is strictly related to the ionic interactions between the arrangement of sulfate groups along the oligomer chain and the alkylammonium ions contained in the eluent phases. Specifically, a better distribution of sulfate groups along the structure ΔU_2S_–A_NS_–G–A_NS3S_ produces a lower steric hindrance than in the ΔU–A_NS6S_–G–A_NS3S_ sequence. Consequently, the former will have a longer retention time than the latter by improving the interaction with the stationary phase of the chromatographic column.

**Figure 5:**
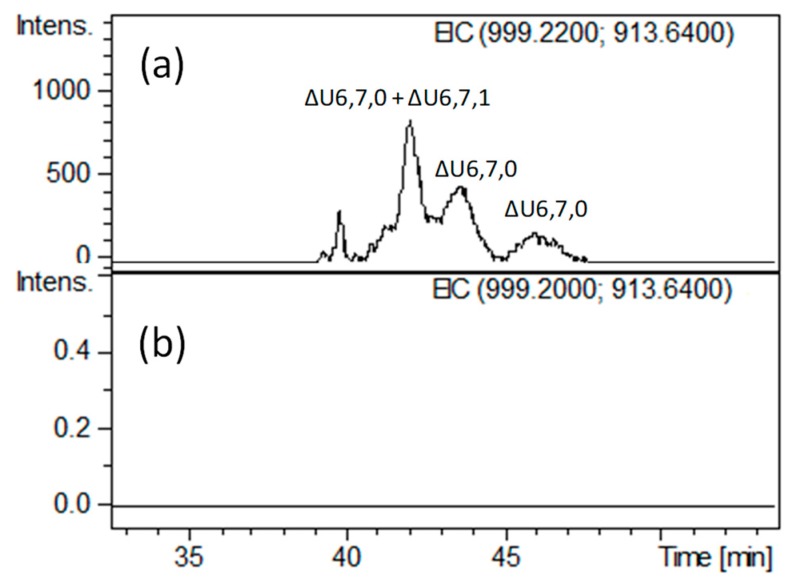
LC-MS profiles obtained by extracted ion chromatogram (EIC) of *m/z* and 999.2 (z = −2) *m/z* 913.6 (z = −2) values attributed to hexasaccharides ΔU6,7,1 ([M + 3DBA]^2–^ ions) and ΔU6,7,0 ([M + 2DBA]^2–^ ions), respectively, (**a**) before and (**b**) after heparanase digestion: (**a**) heparinase digest of BMH; (**b**) heparanase digestion of heparinase digest. The abbreviation system includes, in order, the number of monosaccharide residues, sulfate groups, and *N*-acetyl groups. The symbol ΔU indicates a 4,5-unsaturated uronic acid at the NRE.

Moreover, when applied to hexasaccharides observed in the heparinase-digested HA BMH fraction (ΔU6,7,0 and ΔU6,7,1), the heparanase-based approach may allow to explain their resistance to heparinases. The disappearance of all hexasaccharides after heparanase addition (Figure 5b) and the absence of signals corresponding to pentasaccharides species (monitored by extracted ion chromatograms of mass signals) seem to suggest the hypothesis of two vicinal cleavage sites within the hexasaccharide structures recognized by heparanase. This hypothesis was further supported by the detection of saturated disaccharides such as A2,2,0 and A2,3,0 (Figure S3) consistent with the sequences A_NS3S_–G and A_NS3S6S_–G, respectively, in agreement with the mechanism of action concerning the heparanase enzyme. At any rate, further investigation is needed to clarify their exact structures.

## 3. Materials and Methods

### 3.1. Materials and Reagents

Low-molecular weight heparin dalteparin was from injectable Fragmin^®^ (Pfizer; lot Y08663). BMH was obtained from a commercial supplier (Kin Master, Vila Annes, Brazil). The synthetic pentasaccharide Arixtra^®^ was from GlaxoSmithKline (London, UK). Heparinases I (EC 4.2.2.7), II and III (EC 4.2.2.8) were purchased from Grampian Enzymes (Aberdeen, Scotland, UK), while recombinant human heparanase, covalently linking the 8 and 50 kDa subunits to produce active single-chain heparanase molecules [42], was kindly provided by Israel Vlodavsky. All other reagents and chemicals were of HPLC grade or higher quality.

### 3.2. Isolation of Dalteparin Fraction by SEC-UV

Octasaccharide fraction was isolated from a commercial dalteparin sample using size-exclusion chromatography (SEC) as previously described [9,12]. Briefly, 300 mg sample dissolved in 5 mL of deionized water were loaded on two 5 cm × 90 cm in-series Biogel P6 columns and eluted with 0.25 M ammonium chloride at a flow rate of 1.8 mL/min, using UV detection at 210 nm. The pooled fraction was then desalted using TSK-gel HW40S Toyopearl (Tosoh Bioscience, Yamaguchi, Japan) column 2.6 cm × 60 cm, 10% ethanol at a flow rate of 1.4 mL/min as eluent, and UV detection at 210 nm.

### 3.3. Isolation of a Fraction with High Affinity towards AT(III) from Bovine Mucosal Heparin by Preparative Affinity Chromatography

A 50-mg portion of bovine mucosal heparin were dissolved in 5 mL of the initial eluent A (0.25 M NaCl, 50 mM CH_3_COONH_4_, pH 7.4–7.5) and loaded onto a 70 mL of AT-Sepharose (CNBr-ATIII Sepharose^TM^ 4B) column (2.6 cm × 30 cm) kept at 4 °C and eluted at 2.5 mL/min using the following multistep gradient: 300 mL of eluent A, 390 mL of B (0.5 M NaCl, 50 mM CH_3_COONH_4_, pH 7.4–7.5), 300 mL of eluent C (1 M NaCl, 50 mM CH_3_COONH_4_, pH 7.4–7.5), 300 mL of eluent D (3 M NaCl, 50 mM CH_3_COONH_4_, pH 7.4–7.5). Each eluent was adjusted to pH 7.4–7.5 with 2 M NaOH. Carbazole reaction was used to monitor the elution profile, as described in [43]. The fraction eluted at 3 M NaCl was recovered from the eluent D and desalted as previously described [9].

### 3.4. Heparinases I, II and III Depolymerization

Exhaustive digestion with heparinases I, II and III was performed at 25 °C for 48 h in a total volume of 190 µL, including 20 µL of a 20 mg/mL sample solution in water, 100 µL heparinase I, II and III mixture (0.13 IU/mL of each heparinase in 10 mM monobasic potassium phosphate pH 7.0), and 70 µL of sodium/calcium acetate pH 7 solution (containing 2 mM of calcium acetate and 0.1 mg/mL bovine serum albumin (BSA)). At the end of the incubation time, sample solutions were heated at 100 °C for 2 min to inactivate the enzymes and filtered on a 0.22-µm filter prior to LC-MS analysis.

### 3.5. Heparanase Depolymerization

The heparanase enzymatic activity was tested using a commercially available Arixtra^®^ solution according to the procedure previously described [29]. Dalteparin octasaccharide fraction was digested with heparanase following the same procedure as Arixtra^®^. The heparinases digest of the BMH HA fraction, prepared as specified above, was subjected to heparanase depolymerization using modified procedure including higher enzyme amount. At first, ~10 µg of the heparanase were added to ~268 µg of sample to have an enzyme/substrate ratio of ~1:27 *w/w* (128 µL of heparinases mixture digestion were mixed with 18 µL of a 550 µg/mL heparanase solution in MES buffer solution and 172 µL of 20 mM ammonium acetate containing 2 mM calcium acetate at pH 5.8, in a total volume of 318 µL) and left to stay under stirring for 24 h at 37 °C. Since several resistant tetrasaccharides were still observed, a further 18 µL aliquot of a 550 µg/mL heparanase solution (in MES buffer solution and 172 µL of 20 mM ammonium acetate containing 2 mM calcium acetate at pH 5.8) was added to 223 µL of previous digestion mixture to produce a final enzyme/substrate ratio of 1:11 *w/w* and the reaction was carried out under stirring at 37 °C for other 24 h.

### 3.6. LC-MS Analysis

LC-MS analyses were run on LC system (Platin Blue, Knauer, Berlin, Germany) coupled to ESI-Q-TOF mass spectrometer equipped with an electrospray interface and a high-resolution time-of-flight mass analyzer (Impact II, Bruker Daltonics, Bremen, Germany). Sample solutions (5 µL) were injected on C18 Kinetex column (100 mm × 2.1 mm i.d., with 2.6 µm particles, with precolumn filter, Phenomenex, Aschaffenburg, Germany) held at 35 °C. The mobile phases A (10 mM dibutylamine and 10 mM acetic acid in water) and B (10 mM dibutylamine and 10 mM acetic acid in methanol) were used for the gradient elution.

Dalteparin octasaccharides were separated at the flow rate of 0.3 mL/min applying a multistep gradient: the solvent composition was held at 20% B for the first 5 min, then increased to 53% B over 20 min, and to 70% B over another 1 min, where it was held for 5 min; finally, it was returned to 20% B over 1 min, and held for the last 20 min for equilibrating the chromatographic column. Heparinases digest of the octasaccharide fraction was eluted using the following elution gradient: the solvent composition was held at 10% B for the first 3 min, then increased to 60% B over 30 min, and to 85% B over another 4 min, where it was kept for 8 min and then returned to 10% B over 2 min, and held for other 13 min for column equilibrating. To perform MS/MS analysis, the latter gradient was adjusted for the only purpose of separating two isomeric trisulfated saturated disaccharides. Particularly, mobile phases were run at a flow rate of 0.1 mL/min according to the following gradient: an isocratic step at 10% B for 5 min, followed by a linear gradient from 10% to 35% B in 35 min; then, column washing and reconditioning in the initial conditions were performed.

Heparinases digests of bovine heparin before and after heparanase addition were separated at the flow rate of 0.15 mL/min using the multistep gradient optimized for multicomponent digestion mixture: isocratic step at 10% B for 5 min, followed by a linear gradient from 10% to 80% B in 55 min; then, phase B was increased up to 90% B and kept for 5 min for column washing, followed by a fast linear gradient to restore the initial conditions and column equilibration over 30 min.

The electrospray interface was set in negative ionization mode (Spray Voltage +3500 V), to record full scan mass spectra in the *m/z* 140–2500 mass range. Nitrogen was used as a drying (7 L/min) and nebulizing gas (1.8 bar) and the ion transfer capillary was kept at 200 °C. MS/MS fragmentation experiments were produced by collision-induced dissociation (CID) at 20 eV of collision energy. LC-MS profiles and mass spectra were elaborated using the DataAnalysis software (Bruker Daltonics).

### 3.7. Nomenclature

The abbreviation system used for the oligomers identified by LC-MS includes, in order, the number of monosaccharide residues, sulfate groups, and *N*-acetyl groups. Symbols ΔU, U and A were added to indicate a 4,5-unsaturated uronic acid, a saturated one and a glucosamine unit, respectively, at the NRE. Symbols aM.ol and Rc were used to indicate 2,5-anhydromannitol and contracted ring residues typical for dalteparin [12]. When describing the oligosaccharide sequences, parentheses (e.g., (6S)) indicate possible, but variable between positional isomers, presence of sulfate group in the corresponding position.

Dissociation patterns, produced by collision ion dissociation (CID) technique, are described using the nomenclature of Domon and Costello [44].

## 4. Conclusions

With the increasing need of effective analytical methods aimed to provide full structural characterization of heparin and low-molecular-weight heparin, the introduction of the new heparanase-based strategy, described herein, looks very promising. In fact, the valuable heparinase depolymerization method, which is currently included in numerous analysis schemes of heparins and LMWHs, provides precious structural data but is not sufficient for their full characterization. The introduction of additional studies, including time-consuming isolation of single oligomers, is often requested by the regulatory agencies. We found that the heparanase specificity, used in addition to the existing strategies, represents a potentially powerful approach that can significantly improve elucidation of heparin structural peculiarities by adding another piece to the complex puzzle of heparin full characterization. Given that limited available amounts of pure components represent a major hindrance to structure determination of heparin oligosaccharides, these results, obtained using very a low sample amount, showed a strong potential of heparanase use in developing analytical strategies for heparin structure elucidation allowing to avoid time-consuming intensive procedures of isolation and purification.

It is worth noting that the present strategy used the heparanase depolymerisation step as an additional analytical tool, showing that it may be useful when applied to the samples whose complexity was previously simplified by using other sample preparation methods (e.g., heparinase depolymerization or size-exclusion and/or affinity chromatography) prior to heparanase cleavage. The unambiguous structure assignment of the two major components of a dalteparin octasaccharides fraction allowed to highlight the presence of G–A_NS3S6S_ moieties at their NRE, which is an important structural signature of these drugs, especially in terms of the depolymerization process used in their manufacturing. When applied to bovine heparin oligosaccharides with high affinity for antithrombin, the proposed approach provided relevant structural information about ATBR-related sequences. Specifically, focusing on less abundant components and new positional isomers, the G–A_NS3S_ sequence was expected for many of them. Furthermore, the combination of heparinases and heparanase substrate specificity looks promising for determining additional level of heterogeneity, which is especially useful for comparative studies of these complex drugs. Based on the observed resistance to heparanase of some ATBR-related tetrasaccharides, differences in 6-*O*-sulfation in heparins from different sources should provide different LC-MS profiles of their heparinases–heparanase digests, and are even more sensitive to their structural differences than the traditional heparinase method.

Last, but not least, the obtained data open up new considerations for an enzymatic mechanism of heparanase action which has not yet been fully understood. Despite the declared ability of an endo-hydrolase enzyme to cleave the substrate within the middle of a chain, we observed a favored cleavage at the end (by release of a glucuronic acid). A certain inhibition from G–A_NS3S6S_ intrachain, probably due to a highly sulfated environment, was also unexpected. Further investigations, including kinetics model studies, are required to provide additional insights on the specificity of heparanase towards these substrates.

## Figures and Tables

**Table 1:** MS/MS analysis and ion fragments produced by CID.

*m/z*	z	Fragmentation Ion	
		Isomer No. 1 (U–A_NS3S6S_)	Isomer No. 2 (U_2S_–A_NS6S_)
137.99	−1	^0,2^X_2_ (+1 SO_3_)	^0,2^X_2_ (+1 SO_3_)
168.49	−2	Y_2_ (+2 SO_3_)	Y_2_ (+2 SO_3_)
175.03	−1	B_1_	B_1_
198.99	−1	^0,2^A_2_ (+1 SO_3_)	-
210.99	−1	-	B_1_ – CO_2_ (+1 SO_3_)
222.01	−1	-	Z_2_ – H_2_O (+1 SO_3_)

**Table 2:** Structural hypothesis of detected tetrasaccharides based on heparanase action taking into account both the resistance of a few of them and production of specific enzymatic fragments.

Oligosaccharide Identification by Current Nomenclature	Structure
∆U4,3,1 *	**ΔU–A_NAc6S_–G–A_NS3S_**
**∆U4,4,1** **(1)**	**ΔU–A_NAc6S_–G–A_NS3S6S_**
∆U4,4,1 (2)*	ΔU_2S_–A_NAc6S_–G–A_NS3S_^$^
∆U4,4,0 (1) *	ΔU–A_NS6S_–G–A_NS3S_^$^
∆U4,4,0 (2) *	ΔU_2S_–A_NS_–G–A_NS3S_^$^
**∆U4,5,0** **(1)**	**ΔU–A_NS6S_–G–A_NS3S6S_**
∆U4,5,0 (2) *	ΔU_2S_–A_NS_–G–A_NS3S6S_
∆U4,5,0 (3) *	ΔU_2S_–A_NS6S_–G–A_NS3S_^$^
**∆U4,5,1**	**ΔU_2S_–A_NAc6S_–G–A_NS3S6S_**
∆U4,6,0 *	**ΔU_2S_–A_NS6S_–G–A_NS3S6S_**

**Bold**: previously identified structure [7,10,31,32,33]; * Tetrasaccharides showing some resistance to heparanase action; ^$^ Structures hypothesized by the observed resistance to heparanase action and further supported by the extremely small amount of monomer A1,2,0 (attributed to A_NS3S_).

## Supplementary Materials

The following are available online. Figure S1: Extracted ion chromatograms (EICs) of saturated trisulfated glucosamine A_NS3S6S_ (*m/z* 417.943, z = −1) in the heparinase digests of octasaccharide dalteparin fraction (a) and the same fraction treated with heparanase prior to heparinase cleavage (b), Figure S2: LC-MS profile of bovine HA fraction and mass signal assignment of the main components: expanded chromatogram focused on oligosaccharides region not affected by the heparanase action (monosulfated disaccharides and linkage region fragments), Figure S3: LC-MS profile of bovine HA fraction and mass signal assignment of the main components: expanded chromatogram focused on oligosaccharides region affected by the heparanase action.
