# Supplementary material for: Heparanase as an Additional Tool for Detecting Structural Peculiarities of Heparin Oligosaccharides
Source: Molecules. 2019 Dec 2;24(23):4403. doi: 10.3390/molecules24234403 (PMC6930493; doi:10.3390/molecules24234403)
Supplement: Supplementary file 1 [file molecules-24-04403-s001.pdf]

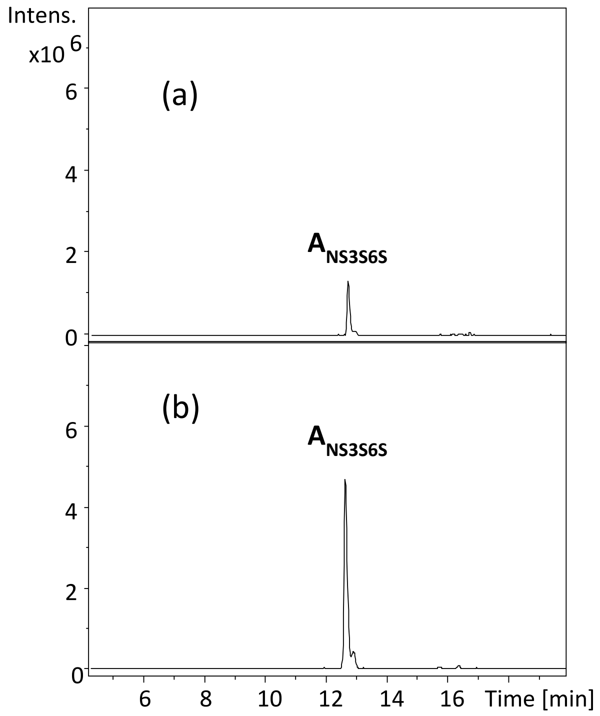

Figure S1: Extracted ion chromatograms (EICs) of saturated trisulfated glucosamine A_{NS3S6S} (*m/z* 417.943, z = -1) in the heparinase digests of octasaccharide dalteparin fraction (a) and the same fraction treated with heparanase prior to heparinase cleavage (b)

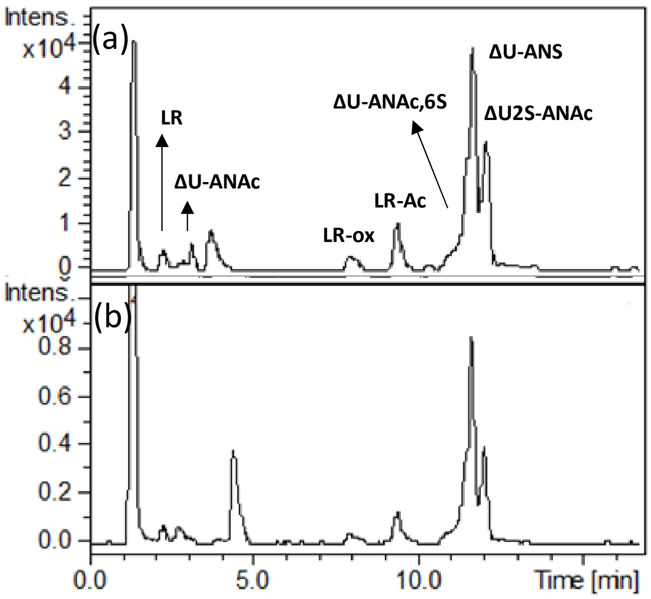

Figure S2. LC-MS profile of bovine HA fraction and mass signal assignment of the main components: expanded chromatogram focused on oligosaccharides region not affected by the heparanase action (monosulfated disaccharides and linkage region fragments)

(a) heparinase digest of BMH, (b) heparanase digestion of heparinase digest.

The abbreviation system includes in order the number of: monosaccharide residues, sulfate groups, and N-acetyl groups. Symbol ΔU was added to indicate a 4,5-unsaturated uronic acid at the NRE. LR, LR-ox, LR-Ac indicate linkage region related structures (see Results and Discussion). #: system peak.

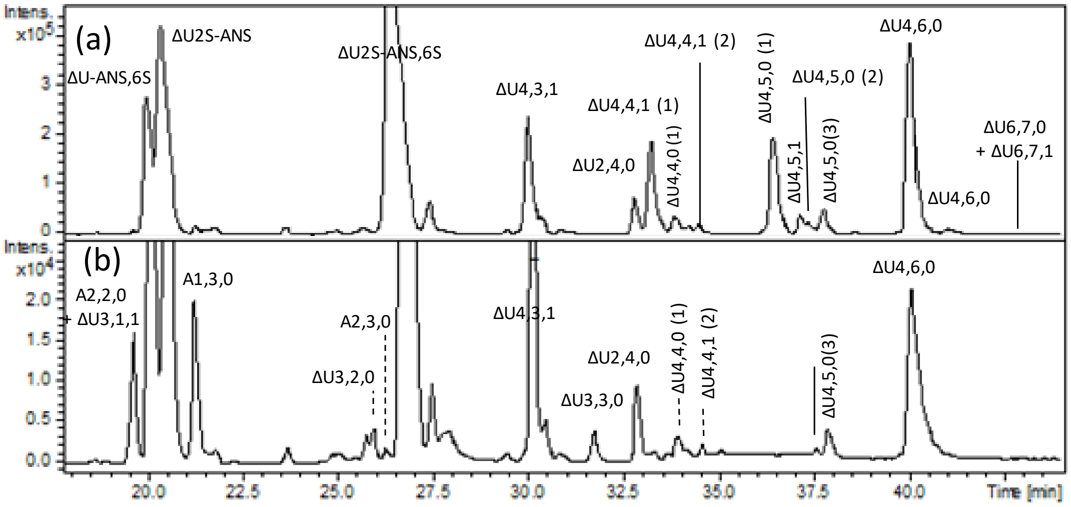

Figure S3. LC-MS profile of bovine HA fraction and mass signal assignment of the main components: expanded chromatogram focused on oligosaccharides region affected by the heparanase action.
(a) heparinase digest of BMH, (b) exhaustive heparanase digestion of heparinase digest.

The abbreviation system includes in order the number of monosaccharide residues, sulfate groups, and N-acetyl groups. Symbols ΔU and A were added to indicate a 4,5-unsaturated uronic acid and a glucosamine unit, respectively, at the NRE.
